# Potential Harm of Prophylactic Platelet Transfusion in Adult Dengue Patients

**DOI:** 10.1371/journal.pntd.0004576

**Published:** 2016-03-25

**Authors:** Tau-Hong Lee, Joshua G. X. Wong, Yee-Sin Leo, Tun-Linn Thein, Ee-Ling Ng, Linda K. Lee, David C. Lye

**Affiliations:** 1 Communicable Disease Center, Institute of Infectious Diseases and Epidemiology, Tan Tock Seng Hospital, Singapore; 2 Yong Loo Lin School of Medicine, National University of Singapore, Singapore; Baylor College of Medicine, UNITED STATES

## Abstract

**Background:**

Thrombocytopenia is a hallmark of dengue infection, and bleeding is a dreaded complication of dengue fever. Prophylactic platelet transfusion has been used to prevent bleeding in the management of dengue fever, although the evidence for its benefit is lacking. In adult dengue patients with platelet count <20,000/mm^3^ without bleeding, we aimed to assess if prophylactic platelet transfusion was effective in reducing clinical bleeding and other outcomes.

**Method:**

We conducted a retrospective non-randomised observational study of dengue patients with platelet count < 20,000/mm^3^ without bleeding (except petechiae) admitted to Tan Tock Seng Hospital from January 2005 to December 2008. Baseline characteristics and clinical outcomes were compared between the non-transfused vs. transfused groups. Outcomes studied were clinical bleeding, platelet increment, hospital length of stay, intensive care unit admission and death.

**Results:**

Of the 788 patients included, 486 received prophylactic platelet transfusion. There was no significant difference in the presence of clinical bleeding in the two groups (18.2% in non-transfused group vs. 23.5% in transfused group; P = 0.08). Patients in the transfused group took a median of 1 day longer than the non-transfused group to increase their platelet count to 50,000/mm^3^ or more (3 days vs. 2 days, P <0.0001). The median duration of hospital stay in the non-transfused group was 5 days vs. 6 days in the transfused group (P< 0.0001). There was no significant difference in the proportion requiring ICU admission (non-transfused 0.66% vs. transfused 1.23%, P = 0.44) and death (non-transfused 0% vs. transfused 0.2%, P = 0.43).

**Conclusion:**

Platelet transfusion in absence of bleeding in adult dengue with platelet count <20,000/mm^3^ did not reduce bleeding or expedite platelet recovery. There was potential harm by slowing recovery of platelet count to >50,000/mm^3^ and increasing length of hospitalization.

## Author Summary

Thrombocytopenia is one of the most prominent clinical features in dengue infection. This may manifest clinically as bleeding, which carries significant morbidity and mortality. For many years, clinicians have given dengue patients platelet transfusion in a bid to increase their platelet counts to prevent hemorrhagic manifestations. However, this practice has not been proven to reduce the risk of bleeding in dengue. Conversely, transfusion of blood products may be detrimental to patients as it can carry risks of fluid overload, transmission of infectious diseases and transfusion reactions. In this study, the authors found that platelet transfusion in the absence of bleeding in adult dengue patients did not prevent bleeding. Instead, this practice is associated with a slower platelet recovery and increased length of hospitalisation. These findings may contribute to better clinical management of thrombocytopenia in dengue patients and limit the use of these precious blood products.

## Introduction

Dengue is estimated to cause 390 million infections annually of which a quarter are symptomatic [1]. In the last 5 decades, the incidence of dengue cases has increased 30 -fold with expanding geographical distribution, leading to major international public health concern[2]. Dengue infection results in a spectrum of clinical syndromes ranging from a mild flu-like illness to life-threatening dengue shock with bleeding and multi-organ failure[3]. The most striking laboratory finding in dengue is thrombocytopenia. Thrombocytopenia was seen in 99% of dengue patients in a study from Trinidad and Tobago [4] and in a study from Taiwan, up to 85% of patients with dengue fever had platelet count of <100,000/mm^3^[5]. Platelet count < 20,000/mm^3^ was found in up to 45% of subjects in the Trinidad and Tobago study [4].

Consequently, platelet transfusion may be a plausible means of preventing hemorrhagic manifestations in dengue fever. Platelet transfusion for thrombocytopenia in dengue fever is a common practice. The proportion of dengue patients receiving platelet transfusion ranged from 7% to 50.3% in studies from Trinidad and Tobago, India, Taiwan and Singapore[4, 6–9]. This wide range reflects varying local practices and general lack of consensus with regards to the management of thrombocytopenia in dengue. In a global survey, 190 out of 306 (62.1%) respondents did not advocate prophylactic platelet transfusion in the absence of bleeding [10]. However, there was also a wide geographic variation in their responses.

A few studies investigated the role of prophylactic platelet transfusion in dengue fever. In a study of pediatric patients with dengue shock syndrome and platelet count of <30,000/mm^3^, preventive transfusion did not reduce bleeding. There was significantly higher fluid balance, incidence of pulmonary edema and increased length of hospital stay associated with preventive transfusion [11]. In an observational cohort study of adult patients with platelet count of <20,000/mm^3^, transfusion of platelets in 188 out of 256 subjects had no effect on incidence of bleeding, rate of platelet increment and length of hospital stay [9]. A restrictive policy adopted in a study from Martinique resulted in platelet transfusion in only 9 out of 350 patients (2.6%). 3 deaths (2 were given platelet transfusion) were reported but they were not related to hemorrhage [12].

We conducted a retrospective observational study to investigate the effect of prophylactic platelet transfusion in the management of thrombocytopenia <20,000/mm^3^ in adult dengue fever and evaluate its usefulness in the prevention of hemorrhagic manifestations.

## Methods

### Study design and patients

We conducted a retrospective analysis of all adult patients who were admitted to Communicable Disease Centre, Tan Tock Seng Hospital, Singapore between January 2005 and December 2008. All patients were tested positive for dengue polymerase chain reaction (PCR) [13] or dengue IgM/IgG (Dengue Duo IgM & IgG Rapid Strip, Panbio Diagnostic, Queensland, Australia) [14] together with probable dengue criteria based on World Health Organization (WHO) 1997 or 2009 dengue guidelines [15,2]. Patients who developed platelet count <20,000/mm^3^, without any bleeding manifestations (with the exception of petechiae) were assessed. Decision to provide or defer prophylactic platelet transfusion was based on clinical judgment of the physicians in-charge. Patients admitted for dengue were managed according to a clinical care pathway developed in Tan Tock Seng Hospital. Clinical data collected were recorded in the pathway.

### Variables and definitions

Data collected from all patients included age, sex, fever duration, medical co-morbidities, signs and symptoms, diagnoses of dengue hemorrhagic fever (DHF) based on WHO 1997 guidelines [15], dengue with warning signs and severe dengue (SD) based on WHO 2009 guidelines [2], hematological and biochemical results, treatment including intravenous fluid, platelet and blood transfusion, progress in hospital including admission to intensive care, length of hospitalization and death.

Patients in the prophylactic platelet transfusion and non-transfusion groups were analyzed for clinical outcomes. Outcomes studied were: i) clinical bleeding which was defined as any bleeding excluding the presence of petechiae, ii)mucosal bleeding from gums, nose or vagina, iii) internal bleeding defined as intracranial, retroperitoneal, gastrointestinal tract bleeding, hemoptysis or hematuria, iv) platelet increment the day after transfusion, v) time for platelet count to exceed 50,000/mm^3^, vi) length of stay (LOS), vii) intensive care unit (ICU) admission, and viii) death.

### Statistical analysis

The chi-squared was used to test for univariate associations between categorical variables. Fisher’s exact test was used if the expected cell frequencies fell below 5. Mann-Whitney U test was used to test for differences in continuous variables. Variables were imputed with their group median if less than 10% of data were missing. Variables with 10% missing data were excluded from the analysis.

Propensity score matching (PSM) was used to analyze the effects of platelet transfusion on bleeding in view of different baseline characteristics in patients who had undergone the treatment regimen. Variables that we believed may influence a clinician's decision to transfuse platelets were included as well. Variables used in the PSM included age, year of infection, DHF, SD, diabetes mellitus, cardiac disease, fever day at presentation, presence of any warning signs, temperature, leucocyte, count neutrophil percentage, platelet count, gender, systolic blood pressure <90mmHg and whether aspartate aminotransferase (AST), prothrombin time (PT) and partial thromboplastin time (PTT) were taken.

Logistic regression was used to identify the risk factors of clinical bleeding. Twenty-six predictors with p<0.2 in the univariate analysis and/or were clinically relevant were entered into the logistic model. Age, gender, Charlson's co-morbidity score [16], hematocrit and whether AST was taken were identified as potential confounders and adjusted for in the model. Patients who did not bleed were randomly split into 4 approximately equal subsets and merged with the cases. Logistic regression was performed on the 4 subsets, and each model was validated against the other 3 subsets. Manual backward elimination was performed to get the most parsimonious model. The results were further validated with stepwise regression using Akaike information criterion. The model with the best average sensitivity and specificity was deemed the most appropriate and further validated with the whole dataset. All statistical analyses were performed using R version 3.0.2 [17] and Stata 13.0 (Stata Corporation, Texas, U.S.A.). All tests were carried out at a 5% significance level.

### Ethics statement

The study was approved by the National Healthcare Group Domain Specific Review Board (DSRB/E/2008/00567) with a waiver of informed consent for the collection of anonymized data.

## Results

Of 7500 patients with dengue fever studied, 788 (10.5%) developed platelet count < 20,000/mm^3^ with no clinical bleeding. Of these, 486 (61.7%) were given prophylactic platelet transfusion. The median volume transfused was 240 ml (range 100-618ml).

The demographic data, dengue severity, symptoms and signs, and laboratory data when patients developed platelet count <20,000/mm^3^ were presented in Table 1. Patients with and without prophylactic platelet transfusion did not differ significantly in terms of gender, median age, co-morbidities and DHF. However, patients who received prophylactic platelet transfusion were more likely to have severe dengue.

**Table 1:** Demographic data, dengue severity, co-morbidities, signs and symptoms and laboratory results of patients with and without prophylactic platelet transfusion.

Variable	Non-transfused (n = 302)	Transfused (n = 486)	*P*
*Demographic*			
Male	219 (72.5%)	356 (73.3%)	0.82
Age, years	40 (22–65)	40 (21–67)	0.79
*Dengue diagnosis at time of platelet count <20*,*000/mm*^*3*^			
Probable dengue 1997/2009	301 (99.7%)	485 (99.8%)	0.40
DHF (Grade 1–4)	44 (14.6%)	83 (17.1%)	0.35
DHF (Grade 1–2)	40 (13.2%)	76 (15.6%)	0.36
Dengue shock syndrome	4 (1.3%)	7 (1.4%)	0.89
Severe dengue^#^	18 (6.0%)	68 (14.0%)	*<0*.*0001*
Severe organ involvement	5 (1.7%)	24 (4.9%)	*0*.*02*
Severe plasma leakage	13 (4.3%)	44 (9.0%)	*0*.*01*
*Co-morbidities*			
Any co-morbidities	75 (24.8%)	128 (26.3%)	0.64
Diabetes mellitus	23 (7.6%)	44 (9.1%)	0.48
Hypertension	39 (12.9%)	76 (15.6%)	0.30
Ischemic heart disease	6 (2.0%)	11 (2.3%)	0.80
*Signs and symptoms at time of platelet count <20*,*000/mm*^*3*^			
Fever duration, days	6 (4–9)	6 (4–8)	*0*.*0002*
Max temperature, °C	37.4 (36.6–38.8)	37.6 (36.6–39.2)	*< 0*.*0001*
Pulse rate	82 (65–103)	85 (65–110)	*0*.*008*
Headache	38 (12.6%)	75 (15.4%)	0.27
Myalgia	66 (21.9%)	112 (23.1%)	0.70
Arthralgia	11 (3.6%)	14 (2.9%)	0.55
Eye pain	2 (0.7%)	5 (1.0%)	0.71
Anorexia	66 (21.9%)	138 (28.4%)	*0*.*04*
Nausea/Vomiting	73 (24.2%)	167 (34.4%)	*0*.*003*
Diarrhea	48 (15.8%)	73 (15.0%)	0.74
Petechiae	66 (21.5%)	91 (18.7%)	0.28
Abdominal tenderness	74 (24.5%)	122 (25.1%)	0.85
Pleural effusion or ascites	2 (0.7%)	5 (1.0%)	0.70
Laboratory results* *at time of platelet count <20*,*000/mm*^*3*^			
Hematocrit, %	44.8 (35.9–52.3)	45.3 (37.3–54.0)	*0*.*049*
Leukocyte count, x 10^3^ leukocytes/mm^3^	3.8 (1.5–9.4)	3.3 (1.4–7.9)	*0*.*0001*
Neutrophils, %	45.2 (20.5–68.2)	50 (25–74)	*< 0*.*0001*
Platelet count, x 10^3^ platelets/mm^3^	16 (10–19)	14 (7–19)	*< 0*.*0001*
AST, U/L	153 (64–657)	207 (64–1496)	*0*.*003*
ALT, U/L	84 (33–349)	117 (32–775)	*0*.*004*
Albumin, g/L	34 (27–41)	35 (27–41)	*0*.*004*

* ranges from 5^th^–95^th^ percentile

^#^ patients with bleeding were excluded at baseline

There were many significant differences in terms of clinical features and laboratory data between the two groups, although absolute differences were minor and unlikely to be of clinical significance. Compared with patients who did not receive prophylactic platelet transfusion, transfused patients had statistically significant shorter duration of fever, higher median temperature and pulse rate; reported more anorexia, nausea and vomiting; had higher median serum hematocrit, neutrophil proportion, serum albumin, AST and ALT, and lower median leukocyte and platelet counts.

With regards to the clinical outcome (Table 2), there was no significant difference in the presence of clinical bleeding in the two groups (18.2% in non-transfused group vs. 23.5% in transfused group; P = 0.08). Surprisingly, the occurrence of internal bleeding after platelet transfusion was slightly more common in transfused patients albeit statistically not significant (1.3% in non-transfused vs. 3.4% in transfused; P = 0.07). Likewise, surprisingly there was more mucosal bleeding after platelet transfusion in transfused (18.5%) versus non-transfused group (9.3%, P<0.001). The median time to any bleeding was 1 day after patients developed platelet count less than 20,000/ mm^3^ (range 1–3 days) with no significant difference between the two groups(P = 0.77). Of those who had clinical bleeding, 91% of patients did so within 48 hours of reaching nadir platelet count regardless of platelet transfusion. 2 patients developed liver failure (platelet transfused) and 1 patient developed renal failure (platelet transfused). There was no patient with pulmonary edema.

**Table 2:** Clinical outcomes of cohort.

Variable	Non-transfused (n = 302)	Transfused (n = 486)	*P*
Clinical outcomes*			
Volume of platelet given (mL)	NA	234 (100–618)	NA
Volume of fluid received (ml) per day	1400 (342–2878)	1530 (500–3000)	<0.01
Received blood transfusion	1	5	0.41
Time to clinical bleeding, days	1 (1–3)	1 (1–3)	0.77
Platelet increment next day, x 10^3^ platelets/mm^3^	5 (-6-31)	8 (-6-43)	< 0.0001
Time for platelet count ≥50 x 10^3^ platelets/mm^3^, days	2 (0–4)	3 (1–5)	< 0.0001
Clinical bleeding, without petechiae	55 (18.2%)	114 (23.5%)	0.08
Internal bleeding	4 (1.3%)	17 (3.4%)	0.07
Mucosal bleeding	28 (9.3%)	89 (18.3%)	0.001
Median length of hospital stay, days	5 (5–7)	6 (4–8)	< 0.0001
Liver failure	0	2	0.53
Renal failure	0	1	1
ICU admission	2 (0.66%)	6 (1.23%)	0.44
Death	0 (0%)	1 (0.2%)	0.43

* ranges are 5^th^–95^th^ percentile

**Fig 1:**
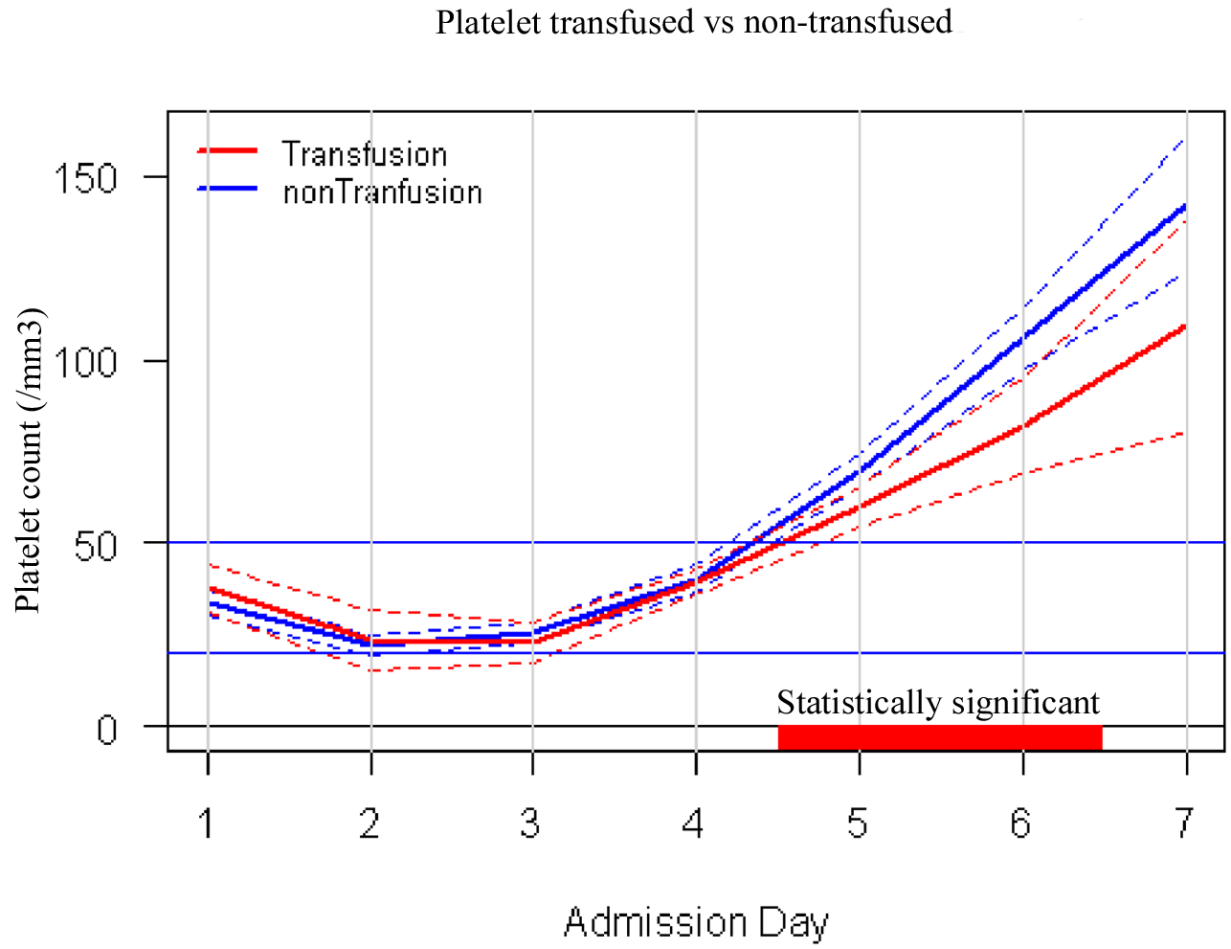
Platelet trend between treatment groups. Patients receiving platelet transfusion took significantly longer time to recover to platelet count of more than 50,000/mm^3^.

While platelet count increased significantly more the next day in the transfused group (by 8,000/ mm^3^) than in the non-transfused group (by 5,000/mm^3^, P<0.0001), patients in the transfused group took a median of 1 day longer than the non-transfused group to increase their platelet count to 50,000/mm^3^ or more (3 days vs. 2 days, P <0.0001). This finding was illustrated in Fig 1 which showed that platelet recovery was slower in the transfusion group. Consequently, the median hospital stay in the non-transfused group was 5 days vs. 6 days in the transfused group (P< 0.0001). There was no significant difference in the proportion requiring ICU admission (non-transfused 0.66% vs. transfused 1.23%, P = 0.44) and death (non-transfused 0% vs. transfused 0.2%, P = 0.43).

**Fig 2:**
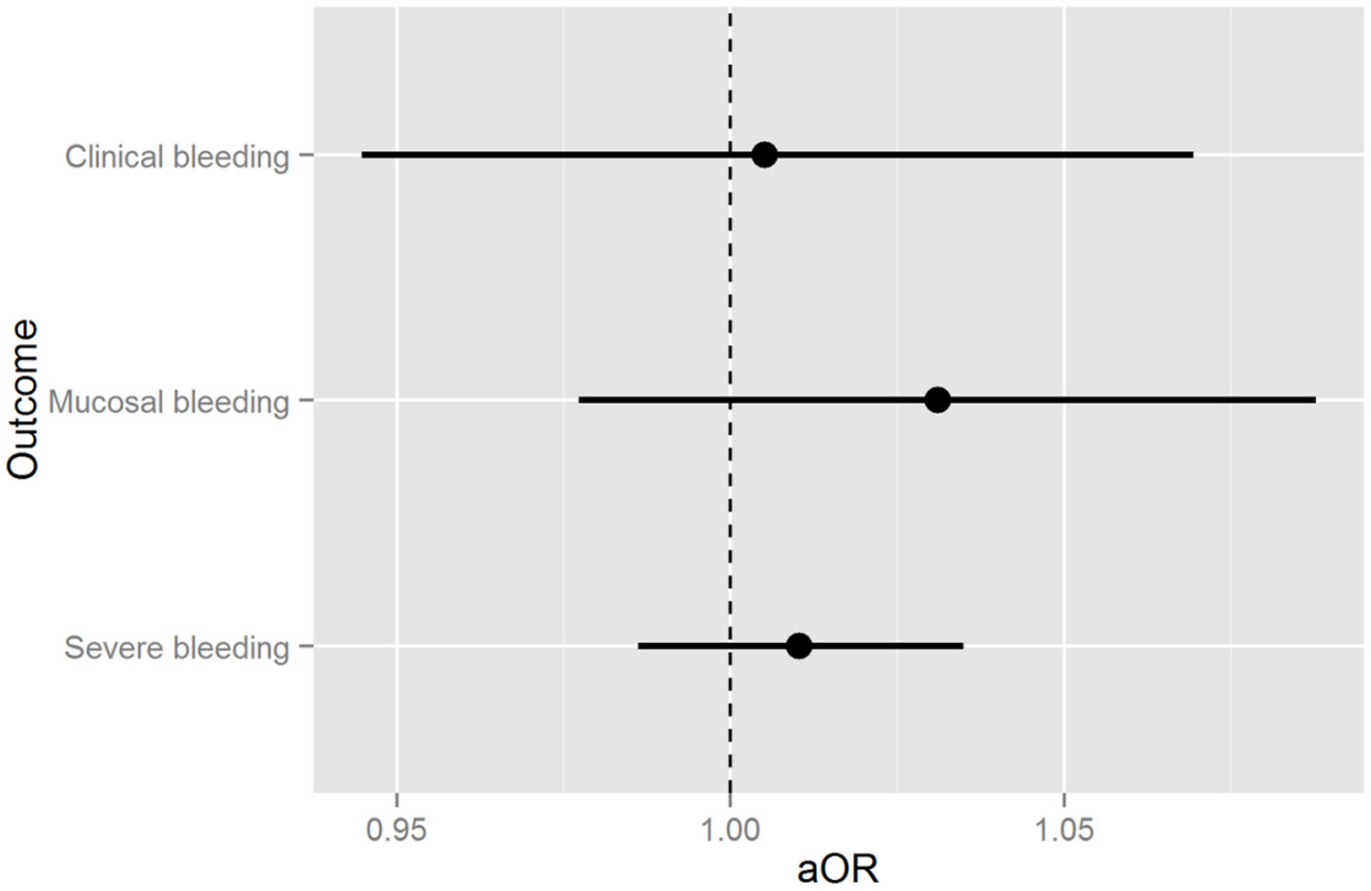
Treatment effects after propensity score matching showing that platelet transfusion had no effect on bleeding after adjustment for variables* that may have influenced physician's decision to transfuse platelets. *Variables: age, year of infection, dengue hemorrhagic fever, severe dengue, diabetes mellitus, cardiac disease, fever day at presentation, presence of any warning signs, temperature, leukocyte count, neutrophil percentage, platelet count, gender, systolic blood pressure < 90mmHg and whether AST, PT and PTT were taken. Clinical bleeding: OR 1.01 (Confidence limit 0.94–1.07); mucosal bleeding: OR 1.03 (Confidence limit 0.98–1.09); internal bleeding: OR 1.01 (Confidence limit 0.97–1.03)

Given the baseline differences, and the excess mucosal bleeding in the transfused group, we performed propensity score matching analysis to examine the effect of prophylactic platelet transfusion and potential biases in treating physicians’ decision to provide prophylactic platelet transfusion. After adjustment for potential confounders of age, year of infection, DHF, SD, diabetes mellitus, cardiac disease, fever day at presentation, presence of any warning signs, temperature, leukocyte count, neutrophil percentage, platelet count, gender, systolic blood pressure < 90mmHg and whether AST, PT and PTT were taken, prophylactic platelet transfusion no longer had any effect in clinical, mucosal and internal bleeding (Fig 2).

We compared the two groups and adjusted for age, gender, dengue severity, Charlson’s co-morbidity score, serum hematocrit and AST. Independent risk factors for any clinical bleeding were presence of fever, lower leukocyte count and higher neutrophil proportion (Table 3).

**Table 3:** Independent risk factors for clinical bleeding in patients with platelet count <20,000/mm^3^.

Symptoms & Signs	aOR (95% CI)
Fever on day of platelet count <20,000/mm^3^	1.84 (1.11–3.05)
Leukocyte count	0.87 (0.76–0.97)
Neutrophils (%)	1.02 (1.004–1.04)

-aOR: Adjusted odds ratio. Adjusted for age, gender, dengue severity, Charlson’s co-morbidity score, hematocrit, and aspartate transaminase.

## Discussion

Thrombocytopenia is common in dengue infection [18] and may be the result of influence from cytokines [19], decreased megakaryopoiesis [20], an increase in the number of dysfunctional megakaryocytes [21], increased destruction of platelets in the liver and spleen [21], destruction of platelets following the binding of dengue-specific antibodies to virus-infected platelets [22, 23] and sequestration of platelets by dengue-infected endothelial cells [24]. Bleeding is a dreaded clinical manifestation of severe dengue. This has long been attributed to thrombocytopenia associated with dengue fever. Prophylactic platelet transfusion has been performed in many instances in an attempt to mitigate this risk.

A 1992 study by American Association of Blood Banks' Transfusion Practice Committee reported that over 70% of hospitals transfused platelets primarily for prophylaxis with an arbitrary threshold of 20, 000/mm^3^ or higher in 80% of these hospitals [25]. This threshold was widely adopted for many years after published clinical data in 1962 despite lack of clinical evidence that 20, 000/mm^3^ was the appropriate transfusion threshold [26]. In a study conducted by Lye et al, no significant relationship was demonstrated between clinical bleeding and platelet count in adult dengue [9]. In our cohort of patients with platelet count <20,000/mm^3^ without any bleeding, prophylactic transfusion was administered in 486 of 788 (61.6%) cases. Our analysis showed that this practice did not reduce bleeding risk in this group of patients who were thought to be at high risk of bleeding.

Platelet transfusion was shown to be associated with slower platelet count recovery with the group receiving transfusion taking one day longer to achieve a platelet count of >50,000/mm^3^. This consequently resulted in an increase in length of stay by one day as platelet recovery to >50,000/mm^3^ was part of the discharge criteria during the study period. Thrombocytopenia in dengue correlated with high thrombopoietin level which stimulated platelet recovery [27]. The transient increase in platelet count from the transfusion could have caused a reduction in serum thrombopoietin level, thereby slowing the endogenous production of platelets from megakaryocytes [28].

Studies suggested that risk factors for bleeding in dengue included degree of thrombocytopenia [29], older age [30], female gender [31], high hematocrit and elevated APTT [32], and high absolute lymphocyte count [31]. While low platelet count was not associated with bleeding in dengue in our study, several correlations for bleeding were identified in our cohort. They were the presence of fever on the day of platelet count <20,000/mm^3^, low white cell count and higher neutrophil proportion. Identification and analysis of other risk factors may contribute to the development of a bleeding risk calculator for the management of dengue patients.

There were limitations to our retrospective study. Firstly, the lack of randomization may have resulted in treatment bias since the decision to transfuse platelets prophylactically was solely based on treating physician's decision. We attempted to correct possible confounders using propensity score matching which confirmed the lack of benefit in platelet transfusion. There were low numbers of severe clinical outcomes such as ICU admission and death in our cohort. Hence the effect of platelet transfusion on these outcomes could not be determined reliably. Secondly, although full blood count, renal and liver panel, PT/APTT were done on admission and FBC was repeated at least daily, further investigations were performed only when clinically indicated.

To our knowledge, our cohort is the largest to date showing a lack of efficacy of prophylactic platelet transfusion in the prevention of bleeding in adult dengue with platelet count <20,000/mm^3^. It resulted in slower platelet recovery and longer hospital stay. We currently reserve platelet transfusion in dengue fever to those with clinically significant bleeding manifestations. This will reduce the use of precious blood products and associated risks of transfusion. Additional research from a randomized trial is needed to address the role of prophylactic platelet transfusion in dengue.
